# Association between antithrombotic treatment and hemorrhagic stroke in patients with atrial fibrillation—a cohort study in primary care

**DOI:** 10.1007/s00228-016-2152-8

**Published:** 2016-11-08

**Authors:** Per Wändell, Axel C Carlsson, Martin Holzmann, Johan Ärnlöv, Sven-Erik Johansson, Jan Sundquist, Kristina Sundquist

**Affiliations:** 1 Division of Family Medicine, Department of Neurobiology, Care Science and Society, Karolinska Institutet, Alfred Nobels Allé 12, 141 83 Huddinge, Sweden; 2 Department of Medical Sciences, Cardiovascular Epidemiology, Uppsala University, Uppsala, Sweden; 3 Department of Emergency Medicine, Karolinska University Hospital, Stockholm, Sweden; 4 Department of Internal Medicine, Solna, Karolinska Institutet, Stockholm, Sweden; 5 School of Health and Social Studies, Dalarna University, Falun, Sweden; 6 Center for Primary Health Care Research, Lund University, Malmö, Sweden

**Keywords:** Atrial fibrillation, Hemorrhagic stroke, Gender, Cardiovascular co-morbidity, Anticoagulants, Mortality

## Abstract

**Objective:**

The objective of this study was to study the association between antithrombotic treatment and risk of hemorrhagic stroke (HS) in patients with atrial fibrillation (AF) treated in primary health care.

**Methods:**

Study population included all adults (*n* = 12,215) 45 years and older diagnosed with AF at 75 primary care centers in Sweden 2001–2007. Outcome was defined as a first hospital episode with a discharge episode of HS after the AF diagnosis. Association between HS and persistent treatment with antithrombotic agents (warfarin, acetylsalicylic acid (ASA), clopidogrel) was explored using Cox regression analysis, with hazard ratios (HRs) and 95 % CIs. Adjustment was made for age, socioeconomic status, and co-morbid cardiovascular conditions.

**Results:**

During a mean of 5.8 years (SD 2.4) of follow-up, 162 patients (1.3 %; 67 women and 95 men) with HS were recorded. The adjusted risk associated with persistent warfarin treatment compared to no antithrombotic treatment consistently showed no increased HS risk, HR for women 0.53 (95 % CI 0.23–1.27) and for men 0.55 (95 % CI 0.29–1.04); corresponding HRs for ASA were, for women, 0.45 (95 % CI 0.14–1.44) and, for men, 0.56 (95 % CI 0.24–1.29).

**Conclusions:**

In this clinical setting, we found no evidence pointing to an increased risk of HS with antithrombotic treatment.

**Electronic supplementary material:**

The online version of this article (doi:10.1007/s00228-016-2152-8) contains supplementary material, which is available to authorized users.

## Introduction

Atrial fibrillation (AF) is the most common heart rhythm disorder in the world [1], affecting around 2 % of the Swedish population [2]. The most important complication in patients with AF is the risk of ischemic stroke, estimated to be five times as common as in individuals without AF [3], with a higher risk among women [4].

Anticoagulant treatment plays a significant role in preventing stroke in AF patients, and anticoagulant (earlier predominantly warfarin) therapy has definite benefits over antiplatelet (mostly acetylsalicylic acid (ASA)) therapy [5]. Given the possible debilitating consequences of stroke and considering the good preventive effect of anticoagulant treatment, it is of great importance to identify individuals with increased risk of stroke among AF patients. Besides, the risk of bleeding complications is a main concern, especially the risk of hemorrhagic stroke (HS) [6]. In general, among stroke patients, 10–20 % have intracerebral bleedings, with a higher risk of functional disability and mortality than ischemic strokes [7].

Among factors of importance of potential anticoagulant-associated hemorrhages are increasing age, prior ischemic stroke, hypertension, and antiplatelet use in addition to anticoagulation [8]. However, in clinical practice complications of warfarin treatment, in general, seem low [9], and the organization of anticoagulation treatment in Sweden, often performed in primary care, seems to contribute to this [10].

The objective of the present study was to explore the risk of first hemorrhagic stroke in men and women in relation to prescription of antithrombotic drugs in a large cohort of AF patients treated in primary health care. We also wanted to explore the mortality risk among AF patients experiencing HS.

## Methods

### Design

This study was performed using individual-level patient data from 75 Swedish primary health care centers (PHCCs), mostly located in Stockholm County (*n* = 48). Men and women with a registered AF diagnosis visiting any of the participating PHCCs between 2001 and 2007 were included in the study. The EPR files of the patients were linked to a database constructed using Swedish national registers (for more information, see Supplementary files). This research database included individual clinical patient data from a total of 1,098,420 subjects registered at these 75 PHCCs. A follow-up was performed using the Swedish Cause of Death Register, which has been shown to be almost complete, 99.8 % [11].

### Study population and co-morbidities

The study included all patients with diagnosed AF, identified by the presence of the ICD-10 code (tenth version of the WHO’s International Classification of Diseases) for atrial fibrillation (I48) in the patients’ medical records. The following cardiovascular-related disorders were used as covariates (see also Supplementary files): hypertension, coronary heart disease (CHD), congestive heart failure (CHF), cerebrovascular diseases (CVDs), and diabetes mellitus. Patients with a first HS during the period were identified, and patients with a first HS before the first AF diagnosis were excluded. In total, 6600 men and 5615 women aged 45 years or older at the time of AF diagnosis and who visited any of the 75 participating PHCCs from January 1, 2001, until December 31, 2007, and with data on neighborhood socioeconomic status were included in the study.

### Outcome variable

The time to first hemorrhagic stroke episode during the assessment period (from registration of first AF diagnosis during 2001–2007 until end of follow-up, December 31, 2010) was defined as having an ICD-10 code (I60–I61) in the Inpatient Register (hospital admissions) or the Cause of Death Register.

Time to mortality from first AF diagnosis to death was registered (until December 31, 2010).

### Demographic and socioeconomic variables

Sex: men and women.

Age was categorized as follows: 45–54, 55–64, 65–74, 75–84, and ≥85 years.

The neighborhood socioeconomic status (SES) areas were categorized into three groups according the neighborhood index: more than one standard deviation (SD) below the mean (high SES or low deprivation level), more than one SD above the mean (low SES or high deprivation level), and within one SD of the mean (middle SES or deprivation level; for more information, see Supplementary files).

Educational attainment was categorized as ≤9 years (partial or complete compulsory schooling), 10–12 years (partial or complete secondary schooling), and >12 years (attendance at college and/or university).

Marital status was characterized as married, unmarried, divorced, or widowed.

### Risk classification of stroke

The stroke risk can be evaluated based on CHADS_2_ or CHA_2_DS_2_-VASc [12].

### Anticoagulant treatment

Prescriptions of antithrombotic treatment, i.e., both anticoagulants and thrombocyte aggregation inhibitors, from 2001 to 2007, were obtained from patient records in primary health care. We studied prescription of warfarin (B01AA03), ASA (B01AC06, B01AC30), and clopidogrel (B01AC04) or ticlopidine (B01AC05). Ticlopidine was categorized in the clopidogrel group. The prescribed antithrombotic drugs were classified as “intention-to-treat” (ITT) if ever present before the years of the first HS or if present at any time among subjects not experiencing a HS [13]. The prescribed warfarin was classified as “per protocol” (PP) if prescribed in the year of the first HS or prescribed among subjects not experiencing a HS at least during half of the years after the first recorded diagnosis of AF or during both 2006 and 2007, presuming a consistent treatment (as no prescription data after 2007 were available).

### Statistical analysis

Baseline characteristics for all included men and women, as well as for those with a recorded first hemorrhagic stroke, were presented as mean (SD) if continuous and as frequencies if categorical.

We classified subjects without and with a first HS according to their CHADS_2_ and CHA_2_DS_2_-VASc scores. We also made stratified analyses in subjects classified as not having a per protocol prescription of warfarin.

We also estimated the incidence rates of a first HS per 100 person-years at risk for men and women. The relative risk of HS for patients on ITT and PP warfarin treatment was analyzed using Cox proportional hazard regression analysis and presented as hazard ratios (HRs) with 95 % confidence intervals (CIs), with the following three models: model 1 age adjusted, model 2 also including socioeconomic factors (educational level, marital status, and neighborhood SES), and model 3 also including cardiovascular co-morbidity. Model specification was tested, and models were checked for interactions. Cox regression was used to estimate mortality risk in patients with an HS, with patients without an HS as referents, with HRs and 95 % CIs.

The study was approved by the regional ethics boards at Karolinska Institutet and Lund University.

## Results

The characteristics of men and women with AF treated in primary care (all, *n* = 12,215) without and with a first recorded HS are shown in Table 1 (data divided by age and by persistent antithrombotic treatment or not in Supplementary Tables 1 and 2, respectively). Mean time to follow-up was 5.77 years (SD 2.41), median follow-up was 5.50 years, and a total of 70,426 person-years at risk were analyzed, i.e., 31,966 among women and 38,460 among men. In total, 162 patients (1.3 %) with HS were recorded, with 67 women (1.2 %) and 95 men (1.4 %).

**Table 1:** Data on women (*n* = 5615) and men (*n* = 6600) aged 45+ years with a diagnosis of atrial fibrillation and without (*n* = 12,121) or with (*n* = 162) a hemorrhagic stroke (HS) in primary care from January 1, 2001, to December 31, 2007

	Women			Men		
	Without HS	With HS		Without HS	With HS	
	*n* = 5548	*n* = 67	*p* value	*n* = 6505	*n* = 95	*p* value
Age (years), mean (SD)	77.1 (9.3)	77.3 (7.2)	0.80	72.1 (10.2)	75.2 (9.0)	0.0032
Age group (years)			0.28			0.067
	*n* (%)	*n* (%)		*n* (%)	*n* (%)	
45–54	104 (1.9)	0 (0.0)		368 (5.7)	1 (1.1)	
55–64	516 (9.3)	4 (6.0)		1205 (18.5)	12 (12.6)	
65–74	1246 (22.5)	16 (23.9)		1994 (30.7)	32 (33.7)	
75–84	2488 (44.8)	37 (55.2)		2287 (35.2)	35 (36.8)	
85+	1194 (21.5)	10 (14.9)		651 (10.0)	15 (15.8)	
Neighborhood SES			0.13			0.25
High	1921 (34.6)	16 (23.9)		2597 (39.9)	41 (43.2)	
Middle	2734 (49.3)	36 (53.7)		2966 (45.6)	46 (48.4)	
Low	893 (16.1)	15 (22.4)		942 (14.5)	8 (8.4)	
Marital status			0.41			0.40
Married	1643 (29.8)	14 (20.9)		3865 (59.7)	62 (65.3)	
Unmarried	391 (7.1)	6 (9.0)		617 (9.5)	8 (8.4)	
Divorced	780 (14.1)	12 (17.9)		1002 (15.5)	9 (9.5)	
Widowed	2708 (49.0)	35 (52.2)		996 (15.4)	16 (16.8)	
Educational level			0.48			0.58
Compulsory school	2561 (52.6)	29 (48.3)		2433 (39.5)	34 (39.5)	
Secondary school	1598 (32.8)	24 (40.0)		2318 (37.6)	36 (41.9)	
College/university	712 (14.6)	7 (11.7)		1410 (22.9)	16 (18.6)	
AF-related disease						
Hypertension	2706 (48.8)	39 (58.2)	0.13	2694 (41.4)	39 (41.1)	0.58
CHD	1156 (20.8)	15 (22.4)	0.76	1314 (20.2)	18 (19.0)	0.76
Heart failure	1140 (20.6)	8 (11.9)	0.082	1127 (17.3)	22 (23.2)	0.14
Valvular disease	272 (4.9)	4 (6.0)	0.57	288 (4.4)	3 (3.2)	0.80
Diabetes mellitus	1076 (19.4)	12 (17.9)	0.76	1284 (19.7)	16 (16.8)	0.48
Drugs						
Ever warfarin	2651 (47.8)	38 (56.7)	0.15	3651 (56.1)	57 (60.0)	0.45
Warfarin ITT	2568 (46.3)	38 (56.7)	0.089	3541 (54.4)	57 (60.0)	0.28
Warfarin PP	2015 (36.3)	23 (34.3)	0.74	2763 (42.5)	42 (44.2)	0.73
Ever ASA	3242 (58.4)	36 (53.7)	0.44	3416 (52.5)	53 (55.8)	0.53
ASA ITT	2863 (51.6)	31 (46.3)	0.39	3007 (46.2)	45 (47.4)	0.83
ASA PP	1780 (32.1)	16 (23.9)	0.15	1924 (29.6)	25 (26.3)	0.49
Ever clopidogrel	195 (3.5)	0	0.17	202 (3.1)	3 (3.2)	0.77
Clopidogrel ITT	141 (2.5)	0	0.42	159 (2.4)	3 (3.2)	0.51
Clopidogrel PP	61 (1.1)	0	1.0	61 (0.9)	1 (1.1)	0.60

Prescription of antithrombotic drug was classified as “intention to treat” (ITT) if ever present before the year of the first stroke or present among subjects not experiencing a stroke and classified as “per protocol” (PP) if present the year before and the year of first stroke or present among subjects not experiencing a stroke if present at least during 3 years, of at least 50 % of actual years after the first recorded year of AF or during both 2006 and 2007

Out of the 67 women with a hemorrhagic stroke, 21 (31 %) were treated with warfarin only, 14 (21 %) were treated with ASA only, 2 (3 %) were treated with a combination of warfarin and ASA, none was treated with clopidogrel, and 30 (45 %) had no antithrombotic treatment at all. Among the 95 men, 34 (36 %) were treated with warfarin only, 17 (18 %) were treated with ASA only, 8 (8 %) were treated with a combination of warfarin and ASA, 1 (1 %) was treated with clopidogrel only, and 35 (37 %) had no antithrombotic treatment at all. No statistically significant increased risk for patients treated with any antithrombotic drug or combination of drugs was found. A recorded HS was present among 37 women (1.0 %) on persistent (per protocol) antithrombotic treatment vs 30 (1.5 %) among women without persistent treatment, with corresponding numbers among men of 60 (1.4 %) and 35 (1.6 %), respectively (division by scores on CHADS_2_ and CHA_2_DS_2_-VASc in Supplementary Tables 3 and 4).

Incident rates for a hemorrhagic stroke per 100 person-years at risk are shown in Table 2, with lower estimates for women than for men, 0.21 vs. 0.25. The age-adjusted relative risk of a hemorrhagic stroke for women as compared to men was HR 0.75 (95 % CI 0.54–1.03). Incident rates were also calculated in subjects without PP antithrombotic treatment, for women 0.28 (95 % CI 0.19–0.40) and for men 0.28 (95 % CI 0.20–0.40) cases per 100 person-years.

**Table 2:** Rate of hemorrhagic stroke (HS) among women and men with atrial fibrillation

	Events at risk (*n*)	Incidence rate (95 % CI)	Warfarin			ASA			All antithrombotic drugs		
			Model 1	Model 2	Model 3	Model 1	Model 2	Model 3	Model 1	Model 2	Model 3
HS											
Women	67/5615	0.210 (0.165–0.266)	0.65 (0.37–1.14)	0.51 (0.22–1.21)	0.53 (0.23–1.27)	0.57 (0.30–1.07)	0.44 (0.14–1.35)	0.45 (0.14–1.44)	0.60 (0.37–0.97)	0.47 (0.22–1.00)	0.48 (0.22–1.04)
Men	95/6600	0.247 (0.202–0.302)	0.81 (0.50–1.31)	0.58 (0.31–1.09)	0.55 (0.29–1.04)	0.60 (0.33–1.08)	0.54 (0.24–1.22)	0.56 (0.24–1.29)	0.76 (0.50–1.16)	0.61 (0.36–1.06)	0.59 (0.34–1.02)

Incidence rate per 100 person-years at risk. Cox regression models for risk of HS in models by “per protocol” analysis (PP) for warfarin and ASA, in comparison with no antitrombotic treatment. Prescription of warfarin (only), ASA (only), or all antithrombotic (AT) drugs was classified as PP if present in the year of first hemorrhagic stroke or present among subjects not experiencing a stroke if present at least during 3 years, of at least 50 % of actual years after first recorded year of AF or during both 2006 and 2007. Model 1 age adjusted, model 2 as model 1 but also adjusted for socioeconomic factors (neighborhood socioeconomic status, educational level, and marital status; for women, also interaction term between neighborhood SES and educational level for warfarin and all AT-drugs, and age and marital status for ASA; for men, also interaction term between age and educational status), and model 3 as model 2 but also adjusted for cardiovascular co-morbidity (hypertension, CHD, CHF, and diabetes; for women, also including interaction terms between age and CHF for warfarin; for men, interaction term between age and educational status)

The risks of a first hemorrhagic stroke with PP treatment with warfarin are shown in men and women in Table 2, with non-significant estimates in fully adjusted models. Among subjects below 65 years of age (women and men combined), we found fully adjusted HRs for PP warfarin, PP ASA, and all PP antithrombotic treatment vs patients without any PP treatment at all of 1.04 (95 % CI 0.79–1.38), 1.63 (95 % CI 0.44–6.04), and 1.14 (95 % CI 0.41–3.14), respectively. The corresponding fully adjusted HRs for subjects 65 years and above were 0.45 (95 % CI 0.26–0.81), 0.35 (95 % CI 0.16–0.78), and 0.45 (95 % CI 0.28–0.75).

When patients with and without persistent antithrombotic treatment were compared, the mean age among women was slightly lower (76.9 vs. 77.5 years) but among men was slightly higher (72.6 vs. 71.2 year) (Supplementary Table 3). For both women and men, patients with persistent antithrombotic treatment vs patients without this were more likely to have hypertension, CHD and diabetes.

The mortality risk for men and women with a first hemorrhagic stroke compared to their counterparts without this was estimated (Table 3), with incidence rates per 100 person-years at risk and HRs by Cox regression. There was a significantly higher risk of mortality in both men and women.

**Table 3:** Risk of mortality among women and men with atrial fibrillation and with or without a hemorrhagic stroke (HS)

	Events/at risk (*n*)	Incidence rate (95 % CI)	Mortality risk by Cox regression (95 % CI)		
			Model 1	Model 2	Model 3
Women					
With HS	40/67	11.31 (8.29–15.41)	1.88 (1.37–2.57)	2.12 (1.51–2.98)	2.25 (1.60–3.16)
Without HS	1923/5548	6.01 (5.75–6.29)	1 (ref)	1 (ref)	1 (ref)
Men					
With HS	48/95	8.82 (6.64–11.70)	1.47 (1.11–1.96)	1.50 (1.10–2.06)	1.46 (1.07–2.01)
Without HS	1935/6505	5.00 (4.78–5.23)	1 (ref)	1 (ref)	1 (ref)

Incidence rate per 100 person-years at risk. Cox regression for mortality risk in women and men with HS. Model 1 age adjusted, model 2 also adjusted for socioeconomic factors (neighborhood socioeconomic status, educational level, and marital status), and model 3 also adjusted for cardiovascular co-morbidity (for women including interaction term between age and CHD)

## Discussion

The main findings of this study were that antithrombotic treatment was not significantly associated with an increased risk of hemorrhagic stroke. Yet, a higher mortality risk among patients that suffered from a hemorrhagic stroke was seen and was especially high among women.

Our findings pointing to a lower risk of HS with anticoagulant treatment were unexpected and quite remarkable. One possible explanation to the unexpected findings could be the misclassification of diagnoses, e.g., that ischemic strokes were classified as hemorrhagic strokes. An initially presented ischemic stroke could have a bleeding component and thus being classified as a bleeding stroke. Another possible explanation could be confounding by indication, i.e., patients regarded as having a higher bleeding risk could be withdrawn from anticoagulant treatment. Besides, we cannot exclude the presence of residual confounding, and the data being used could be of suboptimal accuracy, with possible misclassification of treatment.

In an earlier national Swedish study, the rates of HS were equal in patients treated with warfarin, ASA, or without prophylaxis [14]. Furthermore, another Swedish study found that patients with some contraindication to anticoagulant treatment (dementia, alcohol abuse, renal disease, anemia, any severe bleeding, or frequent falls) but still on warfarin or ASA treatment had a 2–3-fold increased bleeding risk vs patients without contraindications, and the authors of that study concluded that “warfarin-treated patients are highly selected and that decisions not to treat elderly, frail high-risk patients often may be related to complicating co-morbidities and a poor prognosis” [15]. The lower risk estimates for older patients with higher rates of contraindications suggest that these patients are more carefully selected for anticoagulant and antithrombotic treatment, in line with an earlier study [15]. Thus, the clinical decision not to treat frail patients could explain the low risk estimates for HS with antithrombotic treatment.

We confirmed a higher rate of HS among men than in women with AF that has been reported in previous studies [14, 16]. We also confirmed a higher mortality risk among both men and women after hemorrhagic stroke in AF than in those with AF without hemorrhagic stroke [7]. Yet, as far as we know, the present study is the first to show an increased mortality risk of HS among patients with AF treated in primary care.

There are certain limitations of this study. The number of hemorrhagic stroke events was low, and the study may have been underpowered to show an increased risk of anticoagulant treatment. This is an observational study, and prescription of antithrombotic drugs, especially of warfarin, may have been influenced by other factors than those we recorded, i.e., confounding by indication may be one explanation to our results [17]. We did not include subarachnoid hemorrhage [18], which contributes to around 3 % of stroke [19]. Our data were extracted from electronic patient records in primary health care, and data may have been incomplete, e.g., for listings of diagnoses, even if we could expect the diagnoses of cardiovascular diseases and diabetes to be more accurate and complete than many other diagnoses. In contrast, we used hospital data for the diagnosis of the main outcome. In general, the validity of data from hospital registers has been found to be high [20], even if stroke diagnoses seem to be less valid [18, 21]. Besides, severe hemorrhagic strokes could present as sudden death. We had no data available on the type of atrial fibrillation (paroxysmal, persistent, permanent) and rhythm (sinus rhythm, fibrillation). We had no data on some clinical parameters of patients with AF such as electroconversion of AF, catheter ablation, or Cox-Maze operations. Additionally, the HAS-BLED score was not possible to calculate as we had no data on renal or liver function; bleeding history or predisposition, in general; international normalized ratio; or intake of drugs or alcohol [22]. Besides, we did not have access to platelet count and renal function, which are also important predictors of bleeding. Furthermore, we had no data on the non-vitamin K antagonist oral anticoagulant (NOAC) treatments. However, since the variables available in the present study were obtained from primary health care electronic patient records, they may be assumed to mirror the information available for the clinician. As we did not have access to data on time in therapeutic INR range (TIR), we used analyses of PP treatment as attempts in trying to reflect a regular warfarin treatment, and analyses of ITT or not reflect a more crude division. In the statistical analyses, it was not possible to find a balanced model when trying to use propensity score analysis.

Despite the limitations, one of the key strengths of this study is the linkage of clinical data from individual patients to national demographic and socioeconomic data with less than 1 % missing data. The clinical data were also highly complete, and studies using hospital patients only may underestimate the co-morbidity [2]. Another strength is the sample size of the study, i.e., 6600 men and 5615 women and 70,000 person-years at risk analyzed.

In conclusion, our results suggest that anticoagulant and other antithrombotic treatment is safe in clinical practice in primary care with no increased risk of hemorrhagic strokes, suggesting that GPs in Swedish primary care are effective in excluding frail patients at higher risk of bleedings from antithrombotic treatment. Besides, in an earlier study, we found the preventive effect by both warfarin and ASA on ischemic stroke in primary care to be high [13]. The main take-home message is that in this highly selected group of patients, the benefits and risk of warfarin therapy in a country with an excellent track record of warfarin management could be satisfactorily balanced.

## Electronic supplementary material

Table S1 (DOCX 16.9 kb)

Table S2 (DOCX 15.6 kb)

Table S3 (DOCX 13.4 kb)

Table S4 (DOCX 13.7 kb)

Supplementary information (DOCX 12.5 kb)
