# Supplementary material for: Association between antithrombotic treatment and hemorrhagic stroke in patients with atrial fibrillation—a cohort study in primary care
Source: Eur J Clin Pharmacol. 2016 Nov 8;73(2):215–21. doi: 10.1007/s00228-016-2152-8 (PMC5226983; doi:10.1007/s00228-016-2152-8)
Supplement: Supplementary file 1 — (DOCX 16.9 kb) [file 228_2016_2152_MOESM1_ESM.docx]

**Supplementary Table 1** Data on women (n=5,615) and men (n=6,600) aged 45+ years with a diagnosis of atrial fibrillation, divided into subjects younger, or equal to 65 years and above, in primary care from January 1, 2001, to December 31, 2007

|  | Women | |  | Men | |  | Women and men | | |
| --- | --- | --- | --- | --- | --- | --- | --- | --- | --- |
|  | <65 years | ≥65 years |  | <65 years | ≥65 years |  | <65 years | ≥65 years |  |
|  | *n*=624 | *n*=4,991 | p-value | *n*=1,586 | *n*=5,014 | p-value | *n*=2,210 | *n*=10,005 | p-value |
| Age (years), mean (SD) | 58.9 (4.4) | 79.4 (6.9) | <0.001 | 58.0 (4.9) | 76.6 (6.7) | <0.001 |  |  | <0.001 |
| Age group (years) |  |  |  |  |  |  |  |  |  |
|  | *n* (%) | *n* (%) |  | *n* (%) | *n* (%) |  |  |  |  |
| 45–54 | 104 |  |  | 369 |  |  | 473 |  |  |
| 55–64 | 520 |  |  | 1,217 |  |  | 1,737 |  |  |
| 65–74 |  | 1,262 |  |  | 2,026 |  |  | 3,288 |  |
| 75–84 |  | 2,525 |  |  | 2,322 |  |  | 4,847 |  |
| 85+ |  | 1,204 |  |  | 666 |  |  | 1,870 |  |
| Neighborhood SES |  |  | <0.001 |  |  | 0.007 |  |  | <0.001 |
| High | 218 (34.9) | 1,719 (34.4) |  | 647 (40.8) | 1,991 (39.7) |  | 865 (39.1) | 3,710 (37.1) |  |
| Middle | 273 (43.8) | 2,497 (50.0) |  | 679 (42.8) | 2,333 (46.5) |  | 952 (43.1) | 4,830 (48.3) |  |
| Low | 133 (21.1) | 775 (15.5) |  | 260 (16.4) | 690 (13.8) |  | 393 (17.8) | 1,465 (14.6) |  |
| Marital status |  |  | <0.001 |  |  | <0.001 |  |  | <0.001 |
| Married | 331 (53.0) | 1,326 (26.7) |  | 895 (56.6) | 3,032 (60.7) |  | 1,226 (55.6) | 4,358 (43.8) |  |
| Unmarried | 79 (12.7) | 318 (6.4) |  | 282 (17.8) | 343 (6.9) |  | 361 (16.4) | 661 (6.6) |  |
| Divorced | 134 (21.5) | 658 (13.3) |  | 360 (22.8) | 651 (13.0) |  | 494 (22.4) | 1,309 (13.1) |  |
| Widowed | 80 (12.8) | 2,663 (53.6) |  | 44 (2.8) | 968 (19.4) |  | 124 (5.6) | 3,362 (36.5) |  |
| Educational level |  |  | <0.001 |  |  | <0.001 |  |  | <0.001 |
| Compulsory school | 200 (32.4) | 2,390 (55.4) |  | 475 (30.2) | 1,992 (42.6) |  | 675 (30.8) | 4,382 (48.8) |  |
| Secondary school | 265 (43.0) | 1,357 (31.5) |  | 679 (43.2) | 1,675 (35.8) |  | 944 (43.1) | 3,032 (33.7) |  |
| College/university | 152 (24.6) | 567 (13.1) |  | 419 (26.6) | 1,007 (21.5) |  | 571 (26.1) | 1,574 (17.5) |  |
| AF-related disease |  |  |  |  |  |  |  |  |  |
| Hypertension | 252 (40.4) | 2,493 (50.0) | <0.001 | 587 (37.0) | 2,149 (42.9) | <0.001 | 839 (38.0) | 4,642 (46.4) | <0.001 |
| CHD | 65 (10.4) | 1,106 (22.2) | <0.001 | 184 (11.6) | 1,148 (22.9) | <0.001 | 249 (11.3) | 2,254 (22.5) | <0.001 |
| Heart failure | 52 (8.3) | 1,096 (22.0) | <0.001 | 145 (9.1) | 1,004 (20.0) | <0.001 | 197 (8.9) | 2,100 (21.0) | <0.001 |
| Valvular disease | 24 (3.9) | 252 (5.1) | 0.19 | 50 (3.2) | 241 (4.8) | 0.005 | 74 (3.4) | 493 (4.9) | 0.001 |
| Diabetes mellitus | 113 (18.1) | 975 (19.5) | 0.40 | 291 (18.4) | 1,009 (20.1) | 0.12 | 404 (18.3) | 1,984 (19.8) | 0.096 |
| Drugs |  |  |  |  |  |  |  |  |  |
| Ever warfarin | 332 (53.2) | 2,357 (47.2) | 0.005 | 909 (57.3) | 2,799 (55.8) | 0.30 | 1,241 (56.2) | 5,156 (51.5) | <0.001 |
| Warfarin ITT | 322 (51.6) | 2,284 (45.8) | 0.006 | 891 (56.2) | 2,707 (54.0) | 0.13 | 1,213 (54.9) | 4,991 (49.9) | <0.001 |
| Warfarin PP | 253 (40.5) | 1,785 (35.8) | 0.019 | 658 (41.5) | 2,147 (42.8) | 0.35 | 911 (41.2) | 3,932 (39.3) | 0.095 |
| Ever ASA | 252 (40.4) | 3,026 (60.6) | <0.001 | 631 (39.8) | 2,838 (56.6) | <0.001 | 883 (40.0) | 5,864 (58.6) | <0.001 |
| ASA ITT | 233 (37.3) | 2,661 (53.3) | <0.001 | 583 (36.8) | 2,469 (49.2) | <0.001 | 816 (36.9) | 5,130 (51.3) | <0.001 |
| ASA PP | 143 (22.9) | 1,653 (33.1) | <0.001 | 350 (22.1) | 1,599 (31.9) | <0.001 | 493 (22.3) | 3,252 (32.5) | <0.001 |
| Ever clopidogrel | 15 (2.4) | 180 (3.6) | 0.12 | 35 (2.2) | 170 (3.4) | 0.018 | 50 (2.3) | 350 (3.5) | 0.003 |
| Clopidogrel ITT | 12 (1.9) | 129 (2.6) | 0.32 | 31 (2.0) | 131 (2.6) | 0.14 | 43 (2.0) | 260 (2.6) | 0.074 |
| Clopidogrel PP | 5 (0.8) | 56 (1.1) | 0.47 | 13 (0.8) | 49 (1.0) | 0.57 | 18 (0.8) | 105 (1.1) | 0.32 |

Prescription of antithrombotic drug was classified as “intention to treat” (“ITT”) if ever present before the year of the first stroke, or present among subjects not experiencing a stroke; and classified as “per protocol” (“PP”) if present the year before and the year of first stroke, or present among subjects not experiencing a stroke if present at least during three years, of at least 50% of actual years after first recorded year of AF, or during both 2006 and 2007.
