# Supplementary material for: Association between antithrombotic treatment and hemorrhagic stroke in patients with atrial fibrillation—a cohort study in primary care
Source: Eur J Clin Pharmacol. 2016 Nov 8;73(2):215–21. doi: 10.1007/s00228-016-2152-8 (PMC5226983; doi:10.1007/s00228-016-2152-8)
Supplement: Supplementary file 2 — (DOCX 15.6 kb) [file 228_2016_2152_MOESM2_ESM.docx]

Supplementary Table 2. Data on subjects aged 45+ years (n=12,283) with a diagnosis of atrial fibrillation and on any persistent antithrombotic treatment (n=7,961) or not (n=4,322) in primary care from January 1, 2001, to December 31, 2007

|  | Women | |  | Men | |  |
| --- | --- | --- | --- | --- | --- | --- |
|  | With treatment | Without treatment |  | With treatment | Without treatment |  |
|  | *n*=3,587 | *n*=2,028 | p-value | *n*=4,358 | *n*=2,242 | p-value |
| Age (years), mean (SD) | **76.9 (8.5)** | **77.5 (10.5)** | **0.012** | **72.6 (9.5)** | **71.2 (11.3)** | **<0.001** |
| Age group (years) |  |  | **<0.001** |  |  | **<0.001** |
|  | *n* (%) | *n* (%) |  | *n* (%) | *n* (%) |  |
| 45–54 | 33 (0.9) | 71 (3.5) |  | 167 (3.8) | 202 (9.0) |  |
| 55–64 | 326 (9.1) | 194 (9.6) |  | 747 (17.1) | 470 (21.0) |  |
| 65–74 | 870 (24.3) | 392 (19.3) |  | 1,441 (33.1) | 585 (26.1) |  |
| 75–84 | 1,733 (48.3) | 792 (39.1) |  | 1,615 (37.1) | 707 (31.5) |  |
| 85+ | 625 (17.4) | 579 (28.6) |  | 388 (8.9) | 278 (12.4) |  |
| Neighborhood SES |  |  | 0.40 |  |  | 0.35 |
| High | 1,260 (35.1) | 677 (33.4) |  | 1,762 (40.4) | 876 (39.1) |  |
| Middle | 1,749 (48.8) | 1,021 (50.4) |  | 1,986 (45.6) | 1,026 (45.8) |  |
| Low | 578 (16.1) | 330 (16.3) |  | 610 (14.0) | 340 (15.2) |  |
| Marital status |  |  | 0.042 |  |  | 0.058 |
| Married | 1,108 (30.9) | 549 (27.4) |  | 2,646 (60.8) | 1,281 (57.6) |  |
| Unmarried | 244 (6.8) | 153 (7.6) |  | 401 (9.2) | 224 (10.1) |  |
| Divorced | 503 (14.0) | 289 (14.4) |  | 63 (15.2) | 348 (15.7) |  |
| Widowed | 1,729 (48.2) | 1,014 (50.6) |  | 641 (14.7) | 371 (16.7) |  |
| Educational level |  |  | 0.12 |  |  | **0.007** |
| Compulsory school | 1,751 (53.6) | 839 (50.5) |  | 1,691 (40.6) | 776 (37.3) |  |
| Secondary school | 1,055 (32.3) | 567 (34.1) |  | 1,571 (37.7) | 783 (37.7) |  |
| College/university | 463 (14.2) | 256 (15.4) |  | 907 (21.8) | 519 (25.0) |  |
| AF-related disease |  |  |  |  |  |  |
| Hypertension | **1,969 (54.9)** | **776 (38.3)** | **<0.001** | **2,035 (46.7)** | **701 (31.2)** | **<0.001** |
| CHD | **811 (22.6)** | **360 (17.8)** | **<0.001** | **1,031 (23.7)** | **301 (13.4)** | **<0.001** |
| Heart failure | 761 (21.2) | 387 (19.1) | 0.057 | 772 (17.7) | 377 (16.8) | 0.36 |
| Valvular disease | **204 (5.7)** | **72 (3.6)** | **<0.001** | 205 (4.7) | 86 (3.8) | 0.10 |
| Cardiomyopathy | 24 (0.7) | 6 (0.3) | 0.065 | 34 (0.8) | 26 (1.2) | 0.12 |
| CVS | 424 (11.8) | 238 (11.7) | 0.93 | **521 (12.0)** | **196 (8.7)** | **<0.001** |
| HS | 37 (1.0) | 30 (1.5) | 0.14 | 60 (1.4) | 35 (1.6) | 0.55 |
| Diabetes mellitus | **754 (21.0)** | **334 (16.5)** | **<0.001** | **978 (22.4)** | **322 (14.4)** | **<0.001** |
| Drugs |  |  |  |  |  |  |
| Ever warfarin | **2,187 (61.0)** | **502 (24.8)** | **<0.001** | **3,001 (68.9)** | **707 (31.5)** | **<0.001** |
| Warfarin ITT | **2,162 (60.3)** | **444 (21.9)** | **<0.001** | **2,978 (68.3)** | **620 (27.7)** | **<0.001** |
| Warfarin PP | **2,038 (56.8)** | **0 (0.0)** | **<0.001** | **2,805 (64.4)** | **0 (0.0)** | **<0.001** |
| Ever ASA | **2,404 (67.0)** | **874 (43.1)** | **<0.001** | **2,665 (61.2)** | **804 (35.9)** | **<0.001** |
| ASA ITT | **2,169 (60.5)** | **725 (35.8)** | **<0.001** | **2,397 (55.0)** | **655 (29.2)** | **<0.001** |
| ASA PP | **1,796 (50.1)** | **0 (0.0)** | **<0.001** | **1,949 (44.7)** | **0 (0.0)** | **<0.001** |
| Ever clopidogrel | **143 (4.0)** | **52 (2.6)** | **0.005** | **162 (3.7)** | **43 (1.9)** | **<0.001** |
| Clopidogrel ITT | **106 (3.0)** | **35 (1.7)** | **0.004** | **126 (2.9)** | **36 (1.6)** | **0.001** |
| Clopidogrel PP | **61 (1.7)** | **0 (0.0)** | **<0.001** | **62 (1.4)** | **0 (0.0)** | **<0.001** |

Significant differences marked by bold
