# Supplementary material for: Association between antithrombotic treatment and hemorrhagic stroke in patients with atrial fibrillation—a cohort study in primary care
Source: Eur J Clin Pharmacol. 2016 Nov 8;73(2):215–21. doi: 10.1007/s00228-016-2152-8 (PMC5226983; doi:10.1007/s00228-016-2152-8)
Supplement: Supplementary file 3 — (DOCX 13.4 kb) [file 228_2016_2152_MOESM3_ESM.docx]

Supplementary Table 3. First hemorrhagic stroke (HS) (HS) among women (n=5,600) and men (n=6,615) with atrial fibrillation in primary health care during 2001–2010 by scores on CHADS_2_, with number of patients (percentage).

|  | Women | | | |  | Men | | | |
| --- | --- | --- | --- | --- | --- | --- | --- | --- | --- |
| CHADS_2_scores | With  antithrombotic treatment | | Without  antithrombotic treatment | |  | With  antithrombotic treatment | | Without  antithrombotic treatment | |
|  | No HS | HS | No HS | HS |  | No HS | HS | No HS | HS |
|  | n | n (%) | n | n (%) |  | N | n (%) | n | n (%) |
| 0 | 130 | 0 80.0) | 150 | 1 (0.7) |  | 366 | 3 (0.8) | 395 | 3 (0.8) |
| 1 | 759 | 8 (1.0) | 618 | 7 (1.1) |  | 1,225 | 16 (1.2) | 765 | 8 (1.0) |
| 2 | 1,430 | 15 (1.0) | 660 | 13 (1.9) |  | 1,447 | 14 (0.9) | 628 | 17 (2.6) |
| 3 | 855 | 8 (0.9) | 406 | 5 (1.2) |  | 810 | 11 (1.3) | 278 | 3 (1.1) |
| 4 | 284 | 5 (1.7) | 122 | 3 (2.4) |  | 311 | 11 (3.4) | 108 | 2 (1.8) |
| 5 | 80 | 1 (1.2) | 34 | 1 (2.9) |  | 92 | 5 (5.2) | 30 | 2 (6.3) |
| 6 | 12 | 0 (0.0) | 8 | 0 (0.0) |  | 17 | 0 (0.0) | 3 | 0 (0.0) |
| All | 3,550 | 37 (1.0) | 1,998 | 30 (1.5) |  | 4,298 | 60 (1.4) | 2,207 | 35 (1.6) |

Values for patients with or without any antithrombotic treatment by “per protocol”-analysis
