# Supplementary material for: Association between antithrombotic treatment and hemorrhagic stroke in patients with atrial fibrillation—a cohort study in primary care
Source: Eur J Clin Pharmacol. 2016 Nov 8;73(2):215–21. doi: 10.1007/s00228-016-2152-8 (PMC5226983; doi:10.1007/s00228-016-2152-8)
Supplement: Supplementary file 4 — (DOCX 13.7 kb) [file 228_2016_2152_MOESM4_ESM.docx]

Supplementary Table 4. Hemorrhagic stroke (HS) among women (n=5,600) and men (n=6,615) with atrial fibrillation in primary health care during 2001–2010 by scores on CHA_2_DS_2_-VAsc, with number of patients (percentage).

|  | Women | | | |  | Men | | | |
| --- | --- | --- | --- | --- | --- | --- | --- | --- | --- |
|  | With  antithrombotic treatment | | Without  antithrombotic treatment | |  | With  antithrombotic treatment | | Without  antithrombotic treatment | |
|  | No HS | Cer HS | No HS | Cer HS |  | No HS | Cer HS | No HS | Cer HS |
|  | n | n (%) | n | n (%) |  | n | n (%) | n | n (%) |
| CHA_2_DS_2_-VAsc: |  |  |  |  |  |  |  |  |  |
| 0 | – | – | – | – |  | 359 | 3 (0.8) | 392 | 3 (0.8) |
| 1 | 127 | 0 (0.0) | 150 | 1 (0.7) |  | 723 | 7 (1.0) | 431 | 3 (0.7) |
| 2 | 331 | 4 (1.2) | 229 | 3 (1.3) |  | 1,123 | 16 (1.4) | 586 | 12 (2.0) |
| 3 | 823 | 7 (0.8) | 522 | 5 (1.0) |  | 1,096 | 11 (1.0) | 423 | 11 (2.5) |
| 4 | 1,116 | 12 (1.1) | 571 | 15 (2.6) |  | 639 | 8 (1.2) | 237 | 3 (1.3) |
| 5 | 785 | 8 (1.0) | 353 | 1 (0.3) |  | 232 | 10 (4.1) | 107 | 1 (0.9) |
| 6 | 262 | 6 (2.2) | 121 | 4 (3.2) |  | 106 | 5 (4.5) | 25 | 2 (7.4) |
| 7 | 78 | 0 (0.0) | 38 | 1 (2.6) |  | 18 | 0 (0.0) | 5 | 0 (0.0) |
| 8 | 26 | 0 (0.0) | 12 | 0 (0.0) |  | 2 | 0 (0.0) | 1 | 0 (0.0) |
| 9 | 2 | 0 (0.0) | 2 | 0 (0.0) |  | - | - | – | – |
| All | 3,550 | 37 (1.0) | 1,998 | 30 (1.5) |  | 4,298 | 60 (1.4) | 2,207 | 35 (1.6) |

Values for patients with or without any antithrombotic treatment by “per protocol”-analysis
