# Supplementary material for: Association between antithrombotic treatment and hemorrhagic stroke in patients with atrial fibrillation—a cohort study in primary care
Source: Eur J Clin Pharmacol. 2016 Nov 8;73(2):215–21. doi: 10.1007/s00228-016-2152-8 (PMC5226983; doi:10.1007/s00228-016-2152-8)
Supplement: Supplementary file 5 — (DOCX 12.5 kb) [file 228_2016_2152_MOESM5_ESM.docx]

Supplementary information:

*Detailed information of the database:* We used *Extractor* software (<http://www.slso.sll.se/SLPOtemplates/SLPOPage1____10400.aspx>; accessed September 19, 2010) to collect individual files from the electronic patient records (EPR) at the PHCCs. Individual identification numbers were replaced by serial numbers to ensure anonymity.

The EPR files were linked to a database constructed using Swedish national registers, i.e.: the Total Population register (which contains data on, e.g., age and education for the entire population of Sweden); the Inpatient Register (hospital admissions); and the Cause of Death Register. These registers contain individual-level population data for all residents registered in Sweden.

*Identification of comorbidities:* The following diagnoses were included; hypertension (I10–15), coronary heart disease (CHD; I20–25), congestive heart failure (CHF; I50 and I110), cerebrovascular diseases (CVD; I60–69), and diabetes mellitus (E10–14)

*The neighborhood socioeconomic status* *(SES)*: The neighborhood summary index was based on information about female and male residents aged 20 to 64 years, based on the following variables: low educational status (<10 years of formal education); income from all sources, including interest and dividends, that is, <50% of the median individual income); unemployment (excluding full-time students, those completing military service, and early retirees); and receipt of social welfare. We also registered change of neighborhood SES.
